# The Impact of Cognitive Function on the Effectiveness and Safety of Intensive Blood Pressure Control for Patients With Hypertension: A *post-hoc* Analysis of SPRINT

**DOI:** 10.3389/fcvm.2021.777250

**Published:** 2021-11-25

**Authors:** Jiafu Yan, Keyang Zheng, Aoya Liu, Wenli Cheng

**Affiliations:** Hypertension Center, Beijing Anzhen Hospital, Capital Medical University, Beijing, China

**Keywords:** intensive blood pressure control, cognitive function, hypertension, stroke, all-cause mortality

## Abstract

**Background:** Poor cognitive function can predict poor clinical outcomes. Intensive blood pressure control can reduce the risk of cardiovascular diseases and all-cause mortality. In this study, we assessed whether intensive blood pressure control in older patients can reduce the risk of stroke, composite cardiovascular outcomes and all-cause mortality for participants in the Systolic Blood Pressure Intervention Trial (SPRINT) with lower or higher cognitive function based on the Montreal Cognitive Assessment (MoCA) cut-off scores.

**Methods:** The SPRINT evaluated the impact of intensive blood pressure control (systolic blood pressure <120 mmHg) compared with standard blood pressure control (systolic blood pressure <140 mmHg). We defined MoCA score below education specific 25th percentile as lower cognitive function. And SPRINT participants with a MoCA score below 21 (<12 years of education) or 22 (≥12 years of education) were having lower cognitive function, and all others were having higher cognitive function. The Cox proportional risk regression was used to investigate the association of treatment arms with clinical outcomes and serious adverse effects in different cognitive status. Additional interaction and stratified analyses were performed to evaluate the robustness of the association between treatment arm and stroke in patients with lower cognitive function.

**Results:** Of the participants, 1,873 were having lower cognitive function at baseline. The median follow-up period was 3.26 years. After fully adjusting for age, sex, ethnicity, body mass index, smoking, systolic blood pressure, Framingham 10-year CVD risk score, aspirin use, statin use, previous cardiovascular disease, previous chronic kidney disease and frailty status, intensive blood pressure control increased the risk of stroke [hazard ratio (HR) = 1.93, 95% confidence interval (CI): 1.04–3.60, *P* = 0.038)] in patients with lower cognitive function. Intensive blood pressure control could not reduce the risk of composite cardiovascular outcomes (HR = 0.81, 95%CI: 0.59–1.12, *P* = 0.201) and all-cause mortality (HR = 0.93, 95%CI: 0.64–1.35, *P* = 0.710) in lower cognitive function group. In patients with higher cognitive function, intensive blood pressure control led to significant reduction in the risk of stroke (HR = 0.55, 95%CI: 0.35–0.85, *P* = 0.008), composite cardiovascular outcomes (HR = 0.68, 95%CI: 0.56–0.83, *P* < 0.001) and all-cause mortality (HR = 0.62, 95%CI: 0.48–0.80, *P* < 0.001) in the fully adjusted model. Additionally, after the full adjustment, intensive blood pressure control increased the risk of hypotension and syncope in patients with lower cognitive function. Rates of hypotension, electrolyte abnormality and acute kidney injury were increased in the higher cognitive function patients undergoing intensive blood pressure control.

**Conclusion:** Intensive blood pressure control might not reduce the risk of stroke, composite cardiovascular outcomes and all-cause mortality in patients with lower cognitive function.

## Introduction

With aging of the global population, the incidence of hypertension and cognitive impairment has gradually increased, leading to a large social and economic burden (1–3). The results of cross-sectional and longitudinal studies have shown that hypertension is closely associated with impaired cognitive function (4–8). High blood pressure is one of the leading causes of the global burden of disease, and its deleterious effects on the brain contributes to systolic blood pressure-related deaths (9). Elevated blood pressure can lead to poor cognitive function, which might be related to the fact that high blood pressure is a major risk factor for stroke, small vessel disease, focal brain atrophy, and arterial stiffness (10–12).

Previous studies have reported that populations with poor cognitive function have a higher mortality rate. Batty et al. assessed the cognitive function of more than one million men at age 18, and followed them for an average of 20 years; thus, they found a dose-response relationship between mortality and cognitive function with worse cognitive function and a higher risk of mortality (13). In a pooled analyses of data from two prospective stroke cohort studies in Germany and France, individuals with low cognitive function had a significantly higher risk of mortality across the 3-year follow-up (14).

Previous studies found benefits of intensive blood pressure control, including decreasing the risk of primary cardiovascular events and all-cause mortality in the Systolic Blood Pressure Intervention Trial (SPRINT) and stroke in the Action to Control Cardiovascular Risk in Diabetes (ACCORD) blood pressure trial (15, 16). Cognitive function is closely related to blood pressure, and patients with poor cognitive function have worse prognosis. Using data from SPRINT, we explored whether intensive blood pressure control could reduce the risk of stroke, composite cardiovascular outcomes and all-cause mortality in participants with lower or higher cognitive function based on MoCA cut-off scores.

## Materials and Methods

### Data Source and Study Population

We performed a secondary analysis of the SPRINT trial. Data were obtained from the National Institutes of Health Biologic Specimen and Data Repository Information Coordinating Center (https://biolincc.nhlbi.nih.gov/studies/sprint/). We obtained baseline demographic data, laboratory data, physical examination data, baseline Montreal Cognitive Assessment (MoCA) score, prior disease and medication history, and endpoints from the dataset provided by the SPRINT research group. The SPRINT research was conducted in 102 clinical sites in the United States and enrolled 9,361 participants (16), all of whom were randomly assigned to either the intensive treatment group (systolic blood pressure <120 mmHg) or standard treatment group (systolic blood pressure <140 mmHg). All the participants were at least 50 years old and had a systolic blood pressure of 130 mmHg or higher. The populations included in the study must have had at least one of the following increased cardiovascular risk factors: clinical or subclinical cardiovascular disease (CVD), chronic kidney disease (defined as an estimated glomerular filtration rate of 20 to <60 mL/min/1.73 m^2^), Framingham 10-year CVD risk score ≥15% on the basis of laboratory examination in the last 12 months, or age ≥75 years. Patients with type 2 diabetes mellitus, prior stroke, polycystic kidney, and participants with a standing systolic blood pressure of <110 mmHg at baseline were excluded. The intervention was stopped after a median follow-up of 3.26 years owing to a significant reduction in primary cardiovascular events and all-cause mortality in the intensive treatment group compared with that in the standard treatment group (16).

### Baseline Cognitive Function and Frailty Status

All the participants underwent cognitive status screening as assessed by the MoCA at baseline. To correct for racial difference, two points were added to the MoCA score of African Americans and Hispanics (17). We defined MoCA score below education specific 25th percentile as lower cognitive function (17). Therefore, we defined all the SPRINT participants with MoCA scores below 21 (<12 years of education) or 22 (≥12 years of education) as having lower cognitive function (LOWER_CF), and all others as having higher cognitive function (HIGHER_CF).

Baseline frailty status were quantified using SPRINT 37-item frailty index (FI) (17). The frailty status was classified as fit (FI ≥ 0.1), less fit (0.1 < FI ≤ 0.21), and frailty (FI > 0.21).

### Clinical Outcomes and Serious Adverse Events

The primary outcome of our study was stroke. The secondary outcomes included composite cardiovascular outcomes and all-cause mortality. The composite cardiovascular outcomes were the first occurrence of cardiovascular events after randomization, including myocardial infarction (MI), non-MI acute coronary syndrome (non-MI ACS), new-onset stroke, heart failure, and death attributable to CVD. The definition of clinical outcomes was previously published in the SPRINT protocol (18). The secondary outcomes included new-onset stroke and all-cause mortality.

Serious adverse events were defined as fatal or life-threatening events that resulted in a severe or persistent illness requiring hospitalization, prolonged hospitalization, or a medical event determined by the investigator to be a significant hazard or injury to the participant and requiring medical or surgical intervention to prevent injury. Serious adverse events observed in this study included hypotension, syncope, bradycardia, electrolyte abnormalities, injurious falls, and acute kidney injury.

### Statistical Analysis

Categorical variables are expressed as frequencies or percentages. Means ± standard deviations or medians (interquartile ranges) were used for continuous variables based on the distribution of data. We compared the baseline characteristics between intensive and standard blood pressure control in LOWER_CF and HIGHER_CF, respectively. Differences in categorical variables between the treatment arms were evaluated using the Chi-square analysis. The two-tailed *t*-test (normal distribution) or Mann-Whitney *U*-test (skewed distribution) were used to determine any significant differences between the means or medians of the two groups. The normal distribution of data was assessed using a normal Q–Q plot.

To determine whether the benefits of intensive blood pressure control remain robust in different cognitive status, the Cox proportional risk regression was used to compare the clinical outcomes and serious adverse effects of intensive and standard blood pressure control within the lower or higher cognitive functions, respectively. According to the Strengthening the Reporting of Observational studies in Epidemiology (STROBE) statement (19), Model 1 was adjusted for none; Model 2 was adjusted for age (<75 and ≥75 years of age), sex (female, male), ethnicity (black, no black), and body mass index; and Model 3 was further adjusted for age, sex, ethnicity, body mass index, smoking status (never smoked, former smoker, current smoker), baseline systolic blood pressure ( ≤ 132, 132–145, ≥145 mmHg), Framingham 10-year CVD risk score ( ≤ 15%, >15%), aspirin use, statin use, previous CVD, previous chronic kidney disease and frailty status (fit, less fit, frailty). The severity of multicollinearity in Cox model was measured by the variance inflation factor (VIF). If the VIF is ≥5, then multicollinearity existed among variables (Supplementary Table 1).

The robustness of the results in various subgroups [gender, age, ethnicity, systolic blood pressure categories, Framingham 10-year CVD risk, previous CVD, previous chronic kidney disease, baseline aspirin use, statin use and frailty status] were also evaluated by stratified analyses and interaction tests.

All analyses were performed using the statistical software packages R (The R Foundation; http://www.R-project.org) and EmpowerStats (X&Y Solutions, Inc., Boston, Massachusetts, USA; http://www.empowerstats.com). Statistical significance was set at *P* < 0.05.

## Results

### Baseline Characteristics of Included Hypertension Patients

**Figure 1:**
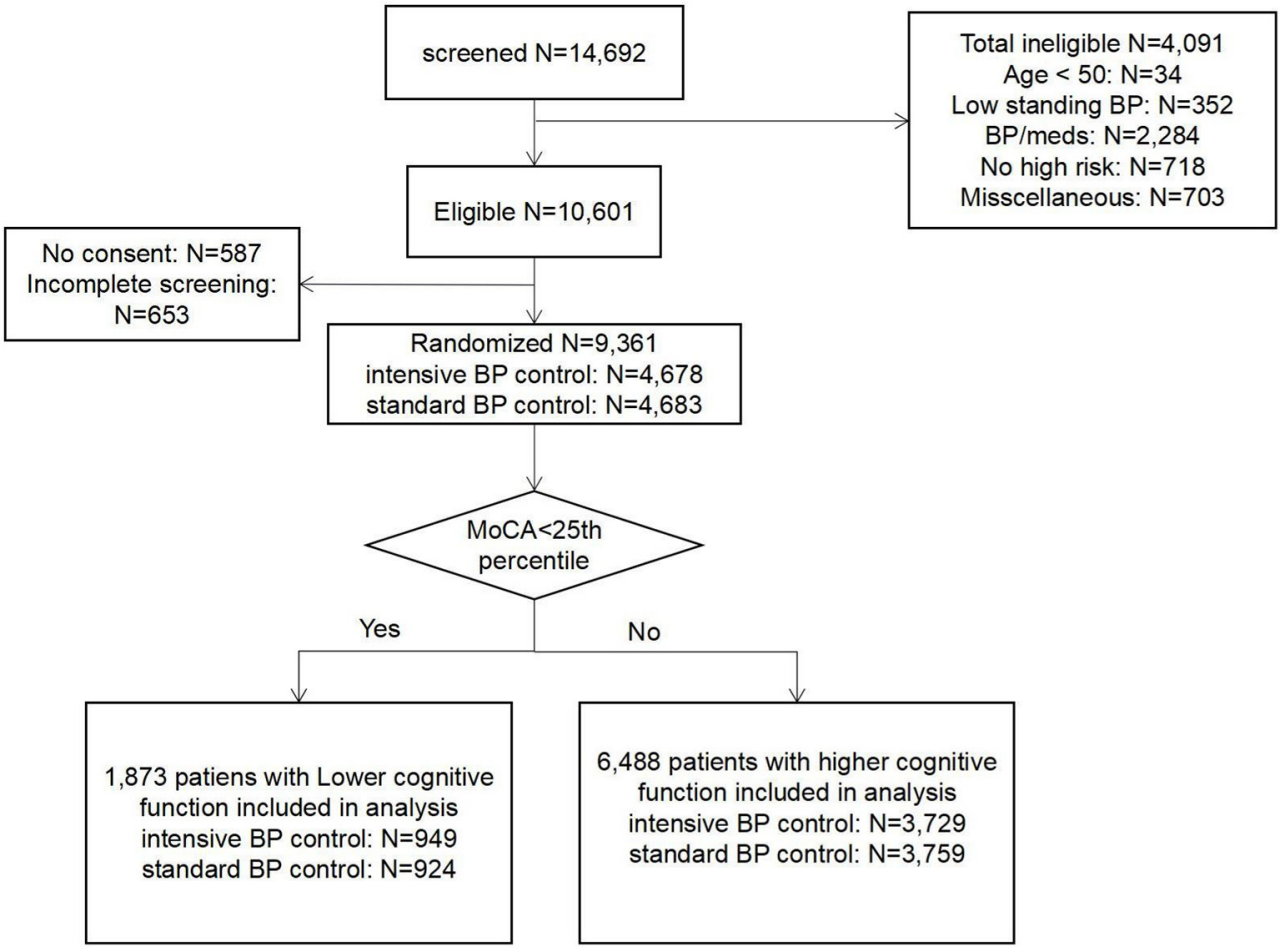
The flow diagram of the study. BP/meds: systolic blood pressure were out of range/ taking too many medications.

The flow diagram of this study is shown in Figure 1. A total of 9,361 participants with hypertension from the SPRINT research were included in the analysis. The median follow-up period was 3.26 years. The mean age for all the participants was 67.92 ± 9.42 years; 3,332 (35.59%) participants were females, 2,802 (29.93%) were black, 1,877 (20.05%) had a history of CVD, and 2,646 (28.27%) had a chronic kidney disease. There were 1,873 patients in the lower cognitive function group and 6,488 in the higher cognitive function group. Supplementary Table 3 showed the baseline differences between patients with lower and higher cognitive function. The baseline data were significantly different between people with lower and higher cognitive function. Patients with lower cognitive function were older, more of the black race, had more frailty, more CVD and CKD and a higher Framingham CVD risk. Table 1 provided the detailed baseline characteristics of all included patients and patients grouped by the treatment arms and two baseline cognitive functions. There was no statistical difference in the baseline data between the intensive and standard blood pressure control in the patients with lower and higher cognitive function, respectively. The frailty status differed between standard and intensive blood pressure control in patients with lower cognitive function.

**Table 1:** Baseline characteristics and outcomes of the study participants according to baseline cognitive function.

	**HIGHER-CF**			**LOWER-CF**			**All participants**
	**Standard BP control**	**Intensive BP control**	***P-*value**	**Standard BP control**	**Intensive BP control**	***P-*value**	
*N*	3,759	3,729		924	949		9,361
MoCA score, median (IQR)	24 (22–27)	24 (22–27)	0.38	17 (15–19)	17 (15–19)	0.36	23 (20–26)
Age, mean (SD), y	66.95 (9.17)	67.09 (9.05)	0.56	71.79 (9.66)	71.25 (9.85)	0.23	67.92 (9.42)
Female, *N* (%)	1,298 (34.53%)	1,334 (35.77%)	0.26	350 (37.88%)	350 (36.88%)	0.66	3,332 (35.59%)
Body mass index, mean (SD)	29.97 (5.76)	30.03 (5.88)	0.66	29.11 (5.49)	29.403 (5.53)	0.25	29.85 (5.77)
**Ethnicity**, ***N*** **(%)**			0.14			0.48	
Black	1,114 (29.64%)	1,047 (28.08%)		309 (33.44%)	332 (34.98%)		2,802 (29.93%)
No black	2,645 (70.36%)	2,682 (71.92%)		615 (66.56%)	617 (65.02%)		6,559 (70.07%)
**Baseline blood pressure, mean (SD)**, **mm Hg**
Systolic	139.42 (15.22)	139.44 (15.56)	0.97	140.63 (16.03)	140.62 (16.55)	0.98	139.67 (15.58)
Diastolic	78.69 (11.90)	78.85 (11.68)	0.55	75.41 (12.04)	75.72 (12.41)	0.58	78.13 (11.94)
Heart rate, mean (SD), bpm	66.24 (11.63)	66.13 (11.39)	0.67	66.35 (12.10)	66.58 (11.97)	0.68	66.24 (11.62)
Urine ACR, median (IQR), mg/g	9.23 (5.48–20.73)	9.35 (5.62–20.00)	0.85	10.66 (5.97–27.10)	10.78 (6.14–28.35)	0.47	9.51 (5.63–21.43)
eGFR, median (IQR), mL/min/1.73 m^2^	71.84 (59.15–85.17)	72.38 (59.13–85.34)	0.47	68.27 (54.36–82.91)	67.83 (53.79–81.68)	0.48	71.37 (58.11–84.68)
Fasting total cholesterol, median (IQR), mg/dL	187 (162–215)	187 (161–215)	0.77	184 (158–211)	186 (159–214)	0.50	187 (161–215)
Fasting HDL cholesterol, mean (SD), mg/dL	52.63 (14.52)	52.82 (14.22)	0.56	53.53 (14.85)	53.40 (14.82)	0.85	52.87 (14.47)
Fasting total triglycerides, median (IQR), mg/dL	107 (78–154)	107 (76–150)	0.53	104 (75–144)	104 (77–141)	0.93	107 (77–150)
Fasting glucose, mean (SD), mg/dL	98.90 (13.31)	98.94 (13.87)	0.89	98.34 (13.61)	98.44 (13.15)	0.87	98.81 (13.55)
Statin use, *N* (%)	1,629 (43.67%)	1,541 (41.58%)	0.07	447 (49.12%)	437 (46.54%)	0.27	4,054 (43.66%)
Aspirin use, *N* (%)	1,866 (49.81%)	1,904 (51.27%)	0.21	484 (52.61%)	502 (53.01%)	0.86	4,756 (50.99%)
**Smoking status**, ***N*** **(%)**			0.98			0.10	
Never smoked	1,623 (43.18%)	1,594 (42.75%)		449 (48.59%)	456 (48.05%)		4,122 (44.03%)
Former smoker	1,622 (43.15%)	1,621 (43.47%)		374 (40.48%)	356 (37.51%)		3,973 (42.44%)
Current smoker	504 (13.41%)	505 (13.54%)		97 (10.50%)	134 (14.12%)		1,240 (13.25%)
Missing data	10 (0.27%)	9 (0.24%)		4 (0.43%)	3 (0.32%)		26 (0.28%)
Frailty index, median (IQR)	0.15 (0.10–0.20)	0.15 (0.10–0.20)	0.78	0.20 (0.15–0.27)	0.22 (0.17–0.27)	0.01	0.16 (0.11–0.22)
**Frailty status**, ***N*** **(%)**			0.95			0.03	
Fit	949 (25.34%)	928 (25.04%)		48 (5.246%)	43 (4.555%)		1,968 (21.14%)
Less fit	1,971 (52.63%)	1,956 (52.78%)		437 (47.760%)	399 (42.267%)		4,763 (51.16%)
Frailty	825 (22.03%)	822 (22.18%)		430 (46.995%)	502 (53.178%)		2,579 (27.70%)
Previous CVD, *N* (%)	707 (18.81%)	684 (18.34%)	0.61	230 (24.89%)	256 (26.98%)	0.30	1,877 (20.05%)
Previous CKD, *N* (%)	995 (26.47%)	985 (26.42%)	0.96	321 (34.74%)	345 (36.35%)	0.47	2,646 (28.27%)
Framingham 10-y CVD risk, median (IQR), %	17.434 (11.705–25.194)	17.005 (11.815–24.680)	0.39	19.692 (12.918–29.118)	19.933 (13.541–29.241)	0.54	17.761 (11.987–25.673)

*eGFR, estimated glomerular filtration rate; HDL, high-density lipoprotein; BMI, body mass index; ACR, albumin creatinine ratio; CVD, cardiovascular diseases; HIGHER_CF, higher cognitive function; LOWER_CF, lower cognitive function; IQR: interquartile range*.

### Relationship Between the Treatment Arms and Clinical Outcomes Within the Lower Cognitive Function and Higher Cognitive Function

We constructed the Cox proportional hazard regression models to estimate the association between the treatment arms and outcomes in the lower cognitive function and higher cognitive function groups, respectively. The results of the three models were listed in Table 2. For the participants with higher baseline cognitive function, the intensive blood pressure control significantly decreased the risk of stroke, composite cardiovascular outcomes and all-cause mortality in all the models; however, hypertensive patients with lower cognitive function might not benefit from the intensive blood pressure control. There was no statistically significant difference in the incidence of composite cardiovascular outcomes and all-cause mortality between the intensive and standard blood pressure control groups. Compared to the standard blood pressure control, patients with lower cognitive function who underwent intensive blood pressure control had a higher risk of stroke. The relationship between the intensive blood pressure control and higher risk of stroke in the lower cognitive function patients was consistent across the three models (Model 1, HR: 1.86, 95% CI: 1.01–3.41, *P* = 0.046; Model 2, HR: 1.86, 95% CI: 1.01–3.41, *P* = 0.045; and Model 3, HR: 1.93, 95% CI: 1.04–3.60, *P* = 0.038). We also investigated the association between intensive blood pressure control and stroke in patients with lower cognitive function based on other MoCA cut-offs (Supplementary Table 2). The lower cognitive function patients (based on other MoCA cutoffs) who underwent intensive blood pressure control had a higher but non-significant risk of stroke. Poor cognitive function still attenuated the benefit of intensive BP control for stroke.

**Table 2:** Stroke, composite cardiovascular outcomes, and all-cause mortality by the treatment arms and baseline cognitive function.

	**Outcomes**	**HR (95%CI)**		
		**Model 1**	**Model 2**	**Model 3**
**Stroke**
LOWER_CF	Standard treatment	1.00	1.00	1.00
	Intensive treatment	1.86 (1.01, 3.41)	1.86 (1.01, 3.41)	1.93 (1.04, 3.60)
	*P-*value	0.046	0.045	0.038
HIGHER_CF	Standard treatment	1.00	1.00	1.00
	Intensive treatment	0.59 (0.38, 0.92)	0.59 (0.38, 0.91)	0.55 (0.35, 0.85)
	*P-*value	0.018	0.017	0.008
**Composite cardiovascular outcomes**
LOWER_CF	Standard treatment	1.00	1.00	1.00
	Intensive treatment	0.89 (0.65, 1.21)	0.88 (0.64, 1.20)	0.81 (0.59, 1.12)
	*P-*value	0.453	0.418	0.201
HIGHER_CF	Standard treatment	1.00	1.00	1.00
	Intensive treatment	0.71 (0.58, 0.86)	0.71 (0.58, 0.86)	0.68 (0.56, 0.83)
	*P-*value	<0.001	<0.001	<0.001
**All-cause mortality**
LOWER_CF	Standard treatment	1.00	1.00	1.00
	Intensive treatment	1.03 (0.71, 1.48)	1.03 (0.71, 1.48)	0.93 (0.64, 1.35)
	*P-*value	0.89	0.88	0.71
HIGHER_CF	Standard treatment	1.00	1.00	1.00
	Intensive treatment	0.63 (0.48, 0.81)	0.64 (0.49, 0.82)	0.62 (0.48, 0.80)
	*P-*value	<0.0001	<0.0001	<0.0001

*HR, hazard ratio; CI, confidence interval; HIGHER_CF, higher cognitive function; LOWER_CF, lower cognitive function*.

*Model 1: adjusted for none. Model 2: adjusted for age, sex, ethnicity, body mass index. Model 3: adjusted for age, sex, ethnicity, body mass. index, smoking status, baseline systolic blood pressure, Framingham 10-year cardiovascular disease risk score, aspirin use, statin use, previous cardiovascular disease, previous chronic kidney disease, frailty status*.

### Subgroup Analyses of Outcomes by Potential Effect Modifiers

**Figure 2:**
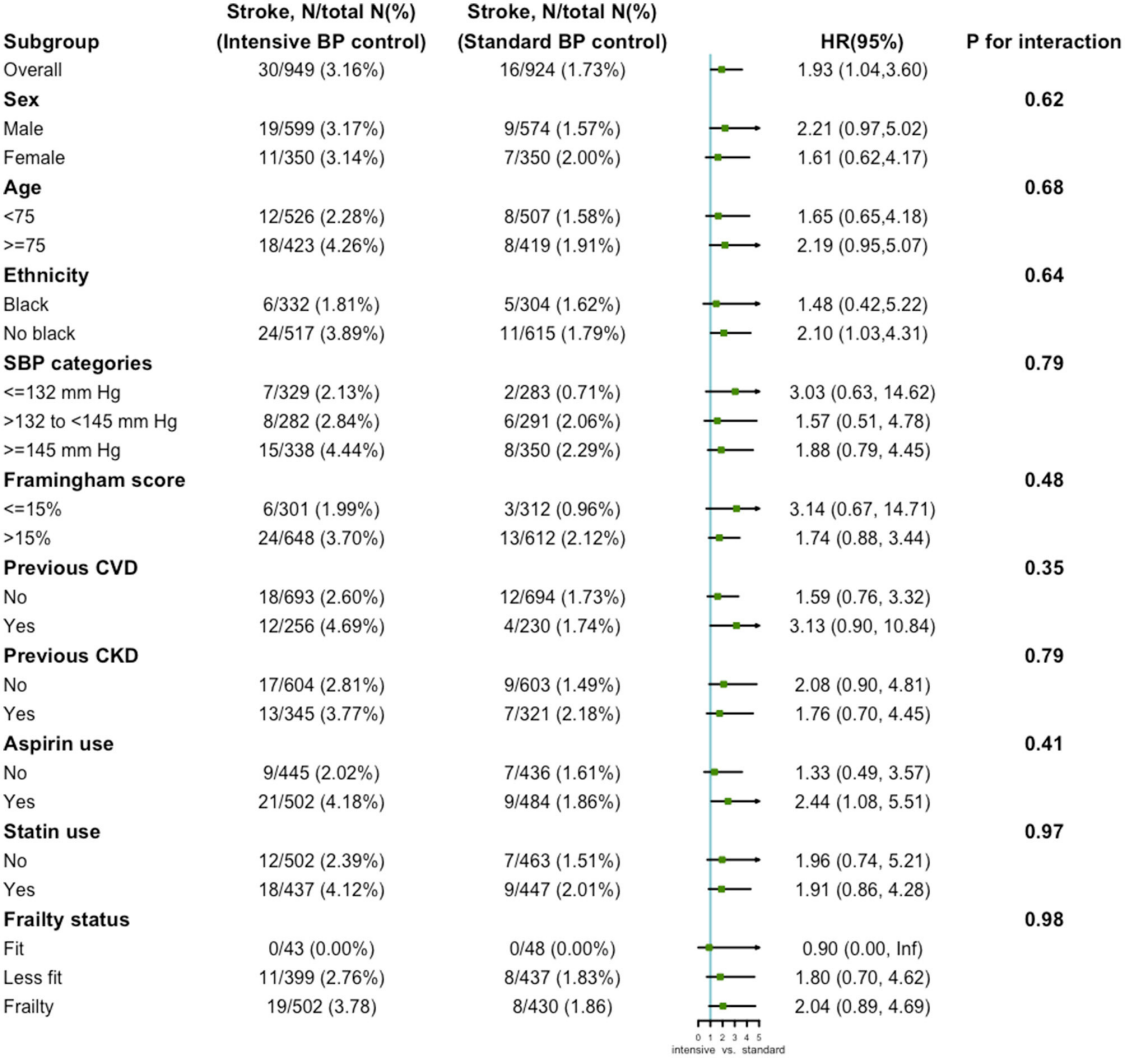
Subgroup analysis of the association between the treatment arms and stroke in lower cognitive function group. Each subgroup analysis was adjusted for all factors in Model 3, except for the stratification factor itself.

Additional interaction and stratified analyses were performed to evaluate the robustness of the association between the treatment arm and risk of stroke in patients with lower cognitive function. Each subgroup analysis was adjusted for all factors in Model 3, except for the stratification factor itself. The results are shown in Figure 2. Generally, intensive blood pressure control was significantly associated with the risk of stroke in patients with lower cognitive function across the pre-specified subgroups. No significant interactions were found between treatment arms and subgroups with respect to stroke.

### Serious Adverse Effects Between Treatment Arms in Lower Cognitive Function

After full adjustment for all covariates in Model 3, the intensive blood pressure control was associated with an increased risk of hypotension (HR: 2.05, 95% CI: 1.03–4.08, *P* = 0.041) and syncope (HR: 1.77, 95% CI: 1.02–3.09, *P* = 0.043) in patients with lower cognitive function. In the higher cognitive function group, intensive blood pressure control could increase the risk of hypotension (HR: 1.66, 95% CI: 1.17–2.35, *P* = 0.004), electrolyte abnormality (HR: 1.53, 95% CI: 1.17–2.01, *P* = 0.002) and acute kidney injury (HR: 1.72, 95% CI: 1.32–2.26, *P* < 0.0001). The results of the relationship between the treatment arms and other serious adverse effects within different cognitive status were shown in Table 3.

**Table 3:** Serious adverse effects by treatment arm and baseline cognitive function.

	**Serious adverse effects**	**HR (95%CI)**		
		**Model 1**	**Model 2**	**Model 3**
**All serious adverse effects**
LOWER_CF	Standard treatment	1.00	1.00	1.00
	Intensive treatment	1.17 (1.01, 1.34)	1.16 (1.01, 1.33)	1.12 (0.97, 1.29)
	*P-*value	0.032	0.040	0.116
HIGHER_CF	Standard treatment	1.00	1.00	1.00
	Intensive treatment	1.01 (0.93, 1.09)	1.01 (0.93, 1.08)	1.00 (0.92, 1.08)
	*P-*value	0.868	0.902	0.924
**Hypotension**
LOWER_CF	Standard treatment	1.00	1.00	1.00
	Intensive treatment	1.98 (1.02, 3.85)	1.96 (1.01, 3.82)	2.05 (1.03, 4.08)
	*P-*value	0.045	0.047	0.041
HIGHER_CF	Standard treatment	1.00	1.00	1.00
	Intensive treatment	1.59 (1.13, 2.24)	1.59 (1.13, 2.25)	1.66 (1.17, 2.35)
	*P-*value	0.008	0.008	0.004
**Syncope**
LOWER_CF	Standard treatment	1.00	1.00	1.00
	Intensive treatment	1.66 (0.97, 2.85)	1.69 (0.98, 2.90)	1.77 (1.02, 3.09)
	*P-*value	0.066	0.058	0.043
HIGHER_CF	Standard treatment	1.00	1.00	1.00
	Intensive treatment	1.22 (0.86, 1.72)	1.22 (0.86, 1.72)	1.21 (0.88, 1.70)
	*P-*value	0.263	0.263	0.288
**Bradycardia**
LOWER_CF	Standard treatment	1.00	1.00	1.00
	Intensive treatment	1.35 (0.75, 2.44)	1.34 (0.74, 2.42)	1.31 (0.72, 2.41)
	*P-*value	0.319	0.333	0.381
HIGHER_CF	Standard treatment	1.00	1.00	1.00
	Intensive treatment	1.13 (0.78, 1.62)	1.12 (0.77, 1.61)	1.10 (0.76, 1.59)
	*P-*value	0.528	0.561	0.620
**Electrolyte abnormality**
LOWER_CF	Standard treatment	1.00	1.00	1.00
	Intensive treatment	1.07 (0.69, 1.65)	1.08 (0.70, 1.67)	1.05 (0.67, 1.63)
	*P-*value	0.769	0.731	0.834
HIGHER_CF	Standard treatment	1.00	1.00	1.00
	Intensive treatment	1.51 (1.16, 1.97)	1.53 (1.17, 1.99)	1.53 (1.17, 2.01)
	*P-*value	0.003	0.002	0.002
**Injurious fall**
LOWER_CF	Standard treatment	1.00	1.00	1.00
	Intensive treatment	1.06 (0.67, 1.70)	1.13 (0.70, 1.82)	1.15 (0.70, 1.87)
	*P-*value	0.818	0.623	0.585
HIGHER_CF	Standard treatment	1.00	1.00	1.00
	Intensive treatment	0.91 (0.66, 1.25)	0.89 (0.64, 1.23)	0.90 (0.65, 1.24)
	*P-*value	0.544	0.466	0.509
**Acute kidney injury**
LOWER_CF	Standard treatment	1.00	1.00	1.00
	Intensive treatment	1.66 (1.06, 2.62)	1.65 (1.05, 2.60)	1.47 (0.93, 2.32)
	*P-*value	0.027	0.030	0.098
HIGHER_CF	Standard treatment	1.00	1.00	1.00
	Intensive treatment	1.65 (1.27, 2.16)	1.69 (1.29, 2.21)	1.72 (1.32, 2.26)
	*P-*value	0.0002	<0.0001	<0.0001

*HR, hazard ratio; CI, confidence interval; HIGHER_CF, higher cognitive function; LOWER_CF, lower cognitive function*.

*Model 1: adjusted for none. Model 2: adjusted for age, sex, ethnicity, body mass index. Model 3: adjusted for age, sex, ethnicity, body mass index, smoking status, baseline systolic blood pressure, Framingham 10-year cardiovascular disease risk score, aspirin use, statin use, previous cardiovascular disease, previous chronic kidney disease, frailty status*.

## Discussion

In this *post-hoc* analysis of SPRINT, we observed that lower cognitive function might attenuate the benefit of intensive blood pressure control for stroke, composite cardiovascular outcomes and all-cause mortality. In old hypertensive patients with lower cognitive function, the intensive blood pressure control could increase the risk of stroke in all models. For patients with higher cognitive function, intensive blood pressure control could reduce the risk of stroke, composite cardiovascular outcomes and all-cause mortality.

A large number of studies have investigated the effect of antihypertensive strategies on CVD and mortality in different populations. The benefits of intensive blood pressure control include decreasing the risk of primary cardiovascular events and all-cause mortality in the SPRINT and stroke in ACCORD blood pressure trial (15, 16). A pooled analysis of SPRINT and ACCORD blood pressure showed that intensive blood pressure reduction did not increase the risk of stroke despite extremely low pulse pressure or mean arterial pressure (20). In a systematic review and meta-analysis of evidence from 19 clinical trials, intensive blood pressure control was associated with a reduced relative risk of major CVD (relative risk reduction: 14%, 95% CI: 4–22%), stroke (relative risk reduction: 22%, 95% CI: 10–32%), and all-cause mortality (relative risk reduction: 9%, 95% CI: −3 to 19%). However, intensive blood pressure control may not benefit hypertensive patients with special conditions. Results from the Action in Diabetes and Vascular Disease-PreterAx and DiamicroN Controlled Evaluation (ADVANCE) trial showed that frailty could attenuate the benefits of intensive blood pressure control for macrovascular and microvascular events (21). A secondary analysis of patients aged 80 years or older in the SPRINT revealed a significant interaction between the baseline cognitive function and treatment arms on the incidence of a composite of primary cardiovascular events and all-cause death (22). The results showed that patients with higher baseline cognitive function could benefit from intensive blood pressure management, with a 60% reduction in the risk of a composite of cardiovascular events and all-cause death. However, those with lower cognitive function had a 33% increased risk (22). We performed a secondary analysis of all participants in the SPRINT study and Cox proportional risk regression for the lower and higher cognitive function groups, respectively. Intensive blood pressure control was significantly associated with a lower risk of stroke, composite cardiovascular outcomes and all-cause death in the patients with higher cognitive function. The participants with lower cognitive function could not derive benefit from the intensive blood pressure control for stroke. However, there was no significant difference in the incidence of composite cardiovascular outcomes and all-cause mortality between the intensive and standard blood pressure control groups in the lower cognitive function group.

In our study, intensive blood pressure control increased the risk of stroke in patients with lower cognitive function. A possible explanation for the increased risk of stroke is that intensive antihypertension might reduce cerebral blood flow. The results of the Secondary Prevention of Small Subcortical Strokes trial showed a J-shaped association between blood pressure and the risk of stroke among patients with recent lacunar infarcts. Systolic blood pressure <120 mmHg and diastolic blood pressure <65 mmHg were associated with an increased risk of stroke (23). It is well-known that chronic hypertension could lead to a blood pressure threshold that maintains cerebral blood flow to shift to a higher level (10, 24). Patients with poor cognitive function are associated with significant cerebral small-vessel diseases, which increase cerebral vascular resistance and disrupt cerebrovascular autoregulation (25). Kim et al. reported that cerebral blood flow velocity decreased in all hypertensive patients after 3 months of antihypertensive treatment, and continued to decrease in hypertensive patients with type-two diabetes mellitus after 6 months of treatment (26). These implied that a higher blood pressure might be critical in maintaining adequate cerebral blood flow and that aggressive blood pressure reduction might worsen cerebral small-vessel diseases. Exaggeration of cerebral small vessel diseases can increase the risk of stroke. Patients with poor cognitive function are often associated with the destruction of cerebral neuronal fiber integrity, which attenuates the sensitivity of arterial baroreflex function (27). Impaired baroreflex function causes fluctuation and instability of blood pressure, resulting in intermittent cerebral hypoperfusion, especially in deep subcortical areas. Intensive blood pressure control may exacerbate this injury in patients with hypertension and lower cognitive function.

This study also found that intensive blood pressure control could not reduce the risk of cardiovascular outcomes and all-cause mortality in patients with lower cognitive function, which conflicted with findings from the SPRINT. This might be mainly attributed to the vulnerability and frailty of patients with lower cognitive function. Several studies have reported that poor cognitive function is associated with a significantly increased risk of mortality (14, 28–31). Patients with poor cognitive function tended to have poor health literacy and less healthy lifestyles (32, 33). A healthy lifestyle could reduce the risk of mortality in patients with poor cognitive function (34, 35). Poor health literacy might attenuate the benefit of intensive blood pressure control for cardiovascular outcomes and mortality. Besides, the lower cognitive function group have a higher rate of frailty which might attenuate the benefit of intensive blood pressure control for all-cause mortality and cardiovascular outcomes (21). Intensive blood pressure control might increase the risk of hypotension and syncope, which could lead to falls. Injurious fall might increase the risk of death. The latest statistics showed an increase in mortality rate attributed to falls among American adults aged 75 years or older (36). However, this study found that in lower cognitive function group, the intensive blood pressure control was associated with a higher risk of hypertension and syncope, but not injurious falls.

Our study had some limitations. The main limitation of our study was that it was a secondary analysis of the SPRINT. Although we adjusted as much as possible for a large number of risk factors that might have altered the clinical outcome, we were not able to adjust for all variables that might affect the outcomes due to the limited database available. Additionally, MoCA was a cognitive screening test and insufficient to reflect true impaired cognitive function. This study was a *post-hoc* analysis of the North American population. The results might not be applicable to other regions and countries because of the impact of race and culture on cognitive assessment.

## Conclusion

In hypertensive patients with lower cognitive function, intensive systolic blood pressure control might increase the risk of stroke, hypotension and syncope. However, composite cardiovascular outcomes and all-cause death did not differ between the treatment arms. Therefore, the management of hypertensive patients with lower cognitive function should be carefully considered, and individualized treatment should be sought.

## Data Availability Statement

Publicly available datasets were analyzed in this study. This data can be found here: https://biolincc.nhlbi.nih.gov/studies/sprint/.

## Author Contributions

JY and KZ statistically analyzed the data and wrote the manuscript. JY, KZ, and AL prepared the figures and tables. WC provided the ideas and revised the manuscript. All the authors agree with the published version of the manuscript.

## Funding

This study was supported by the Capital Health Development Scientific Research Project 2020-2-2064.

## Conflict of Interest

The authors declare that the research was conducted in the absence of any commercial or financial relationships that could be construed as a potential conflict of interest.

## Publisher's Note

All claims expressed in this article are solely those of the authors and do not necessarily represent those of their affiliated organizations, or those of the publisher, the editors and the reviewers. Any product that may be evaluated in this article, or claim that may be made by its manufacturer, is not guaranteed or endorsed by the publisher.
