# Supplementary material for: The Impact of Cognitive Function on the Effectiveness and Safety of Intensive Blood Pressure Control for Patients With Hypertension: A post-hoc Analysis of SPRINT
Source: Front Cardiovasc Med. 2021 Nov 25;8:777250. doi: 10.3389/fcvm.2021.777250 (PMC8655835; doi:10.3389/fcvm.2021.777250)
Supplement: Supplementary file 1 [file Table_1.DOCX]

**Table S1. Multicollinearity test for three Cox models**

| Variables | Outcomes (the variance inflation factor in three Cox models) | | |
| --- | --- | --- | --- |
|  | Stroke | Composite outcome | All-cause mortality |
| Treatment arm | 1 | 1 | 1 |
| age | 1.3 | 1.3 | 1.3 |
| sex | 1.4 | 1.4 | 1.4 |
| ethnicity | 1.1 | 1.1 | 1.1 |
| Body mass index | 1.1 | 1.1 | 1.1 |
| Systolic blood pressure | 1.2 | 1.2 | 1.2 |
| Smoking status | 1.2 | 1.2 | 1.2 |
| Aspirin use | 1.1 | 1.1 | 1.1 |
| Statin use | 1.3 | 1.3 | 1.3 |
| Previous CVD | 1.2 | 1.2 | 1.2 |
| Previous CKD | 1.7 | 1.7 | 1.7 |
| Framingham 10-y CVD risk | 1.2 | 1.2 | 1.2 |
| Frailty status | 1.2 | 1.2 | 1.2 |

# For multicollinearity test, we included adjustment for treatment arm (intensive versus standard blood pressure control), age (< 75 and ≥ 75 years of age), sex (female, male), ethnicity (black, no black), body mass index; smoking status (never smoked, former smoker, current smoker), baseline systolic blood pressure (≤ 132, 132 to 145, ≥ 145 mmHg), Framingham 10-year CVD risk score (≤ 15%, > 15%), aspirin use, statin use, previous CVD, previous chronic kidney disease and frailty status (fit, less fit, frailty). If variance inflation factor ≥ 5, multicollinearity existed among variables.

Table S2. Intensive blood pressure control and stroke in lower cognitive function based on different MoCA cutoffs.

| Lower cognitive function | Stroke No./total No. of patients(%) | |  |  |
| --- | --- | --- | --- | --- |
| (based on different MoCA cut-offs) | Intensive blood pressure control | Standard blood pressure control | HR(95%CI) | P value |
| MoCA≤17 | 8/266 (3.00%) | 2/253 (0.70%) | 3.57 (0.69, 18.48) | 0.129 |
| MoCA≤19 | 18/507 (3.5%) | 7/479 (1.4%) | 2.29 (0.95, 5.54) | 0.067 |
| MoCA≤21 | 30/949 (3.16%) | 16/924 (1.73%) | 1.93 (1.04, 3.60) | 0.038 |
| MoCA≤23 | 36/1617 (2.2%) | 32/1647 (1.9%) | 1.18 (0.73, 1.91) | 0.502 |
| MoCA≤25 | 47/2518 (1.8%) | 44/2571 (1.7%) | 1.08 (0.72, 1.64) | 0.703 |

#To correct for racial differences, 2 points were added to the scores of African-American and Hispanic participants. To control for educational status, we added 1 point to the MoCA score in patients with <12 years of education.

**Table S3. Baseline characteristics of the study participants according to baseline cognitive function**

|  | **Higher cognitive function** | **Lower cognitive function** | **P value** | **All participants** |
| --- | --- | --- | --- | --- |
| N | 7488 | 1873 |  | 9361 |
| MoCA score, median(IQR) | 24 (22-27) | 17 (15-19) | <0.001 | 23（20-26） |
| Age, mean(SD), y | 67.02 (9.11) | 71.52 (9.76) | <0.001 | 67.92（9.42） |
| Female, N(%) | 2632 (35.15%) | 700 (37.37%) | 0.072 | 3332 (35.59%) |
| Body mass index, mean(SD) | 30.00 (5.82) | 29.26 (5.51) | <0.001 | 29.85（5.77） |
| **Ethnicity, N(%)** |  |  | <0.001 |  |
| Black | 2161 (28.86%) | 700 (37.37%) |  | 2802 (29.93%) |
| No black | 5327 (71.14%) | 1232 (65.78%) |  | 6559 (70.07%) |
| **Baseline blood pressure, mean(SD)**, **mm Hg** |  |  |  |  |
| Systolic | 139.43 (15.39) | 140.63 (16.29) | 0.003 | 139.67（15.58） |
| Diastolic | 78.77 (11.79) | 75.57 (12.23) | <0.001 | 78.13（11.94） |
| Heart rate, mean(SD), bpm | 66.19 (11.51) | 66.47 (12.03) | 0.350 | 66.24（11.62） |
| Urine ACR, median(IQR), mg/g | 9.30 (5.56-20.36) | 10.71 (6.07-27.66) | <0.001 | 9.51(5.63-21.43) |
| eGFR, median(IQR), mL/min/1.73 m^2^ | 72.09 (59.13-85.27) | 68.08 (54.08-82.05) | <0.001 | 71.37（58.11-84.68） |
| Fasting total cholesterol, median(IQR), mg/dL | 187.00 (161.00-215.00) | 185.00 (159.00-213.00) | 0.009 | 187（161-215） |
| Fasting HDL cholesterol, mean(SD), mg/dL | 52.72 (14.37) | 53.46 (14.83) | 0.050 | 52.87（14.47） |
| Fasting total triglycerides, median(IQR), mg/dL | 107.00 (77.00-152.00) | 104.00 (76.00-142.00) | 0.005 | 107（77-150） |
| Fasting glucose, mean(SD), mg/dL | 98.92 (13.59) | 98.39 (13.37) | 0.135 | 98.81（13.55） |
| Statin use, N (%) | 3170 (42.63%) | 884 (47.81%) | <0.001 | 4054 (43.66%) |
| Aspirin use, N (%) | 3770 (50.54%) | 986 (52.81%) | 0.079 | 4756 (50.99%) |
| **Smoking status, N(%)** |  |  | <0.001 |  |
| Never smoked | 3217 (42.96%) | 905 (48.32%) |  | 4122 (44.03%) |
| Former smoker | 3243 (43.31%) | 730 (38.97%) |  | 3973 (42.44%) |
| Current smoker | 1009 (13.47%) | 231 (12.33%) |  | 1240 (13.25%) |
| Missing data | 19 (0.25%) | 7 (0.37%) |  | 26 (0.28%) |
| Frailty index, median(IQR) | 0.15 (0.10-0.20) | 0.21 (0.16-0.27) |  | 0.16 (0.11-0.22) |
| **Frailty status, N(%)** |  |  | <0.001 |  |
| Fit | 1877 (25.19%) | 91 (4.90%) |  | 1968 (21.14%) |
| Less fit | 3927 (52.70%) | 836 (44.97%) |  | 4763 (51.16%) |
| Frailty | 1647 (22.10%) | 932 (50.13%) |  | 2579 (27.70%) |
| Previous CVD, N (%) | 1391 (18.58%) | 486 (25.95%) | <0.001 | 1877 (20.05%) |
| Previous CKD, N (%) | 1980 (26.44%) | 666 (35.56%) | <0.001 | 2646 (28.27%) |
| Framingham 10-y CVD risk, median (IQR), % | 17.17 (11.75-24.98) | 19.85 (13.30-29.17) | <0.001 | 17.761 (11.987-25.673) |

eGFR: estimated glomerular filtration rate; HDL: high-density lipoprotein; BMI: body mass index; ACR: albumin creatinine ratio; CVD: cardiovascular diseases; HIGHER_CF: higher cognitive function; LOWER_CF: lower cognitive function; IQR: interquartile range.
